# Internet use patterns among older adults with different frailty statuses: a qualitative user persona study from a health equity perspective

**DOI:** 10.3389/fpubh.2026.1912591

**Published:** 2026-10-08

**Authors:** Keke Ren, Shan Li, Lin Li, Yue Xiang, Xiling Yang, Qinghua Zhao, Huanhuan Huang

**Affiliations:** 1 Department of Nursing, The First Affiliated Hospital of Chongqing Medical University, Chongqing, China; 2 Nursing Research Center, The First Affiliated Hospital of Chongqing Medical University, Chongqing, China

**Keywords:** frailty, health equity, internet use, older adults, qualitative research, user personas

## Abstract

**Objective:**

This study aimed to explore patterns of internet use and related influencing factors among older adults with varying frailty statuses from a health equity perspective by constructing user personas, thereby providing a basis for developing targeted digital inclusion strategies.

**Methods:**

A descriptive qualitative design was employed. From October to December 2025, 24 older adults with internet-use experience were recruited from one community hospital and one tertiary hospital in Chongqing, China, using purposive sampling combined with a maximum variation strategy. Frailty status was assessed using the Frailty Index, and data were collected through face-to-face semi-structured interviews. Data were analyzed using inductive content analysis. Individualized multidimensional profiles were constructed for each participant and subsequently compared across cases to develop user personas. The three-level digital divide framework was then applied as an interpretive lens to compare the identified personas.

**Results:**

Four core dimensions were identified: health status and self-management, digital participation foundation and capability, internet-use orientation and behavioral patterns, and constraints and enabling conditions. Based on these dimensions, five user personas were developed: Health-Empowered Integrators, Basic-Function Adapters, Entertainment-Oriented Users, Resource-Limited Users, and Health-Affected Users. The personas differed in internet access, digital capability and actual patterns of use, and opportunities to translate digital participation into health-related resources and potential benefits. Frailty was an important health-related context influencing digital participation, but it did not correspond directly to patterns of internet use. Instead, health-related functional limitations, digital capability, educational and learning resources, and social support jointly contributed to different patterns of digital participation.

**Conclusion:**

Internet use among older adults is heterogeneous. Patterns of digital participation are not determined by frailty severity alone but are jointly influenced by health, capability, and resource-related conditions. Providing targeted support according to the characteristics of different user personas may help promote more equitable digital participation and access to health-related digital resources among older adults.

## 1 Introduction

Global population aging has become increasingly pronounced. The number of people aged 60 years and older worldwide is projected to increase from 1 billion in 2020 to 1.4 billion in 2030, and to reach approximately 2.1 billion by 2050 (1). Population aging has therefore become an important public health and social challenge worldwide. China is also experiencing an accelerated aging process (2). At the same time, the country's level of digitalization continues to rise. According to the 57th Statistical Report on China's Internet Development, as of December 2025, China's internet penetration rate had reached 80.1%, and the internet use rate among people aged 60 years and older had reached 53.7%, indicating that more older adults are gaining access to digital technologies and participating in digital society (3). For older adults, the internet not only facilitates access to information and health-related resources but also provides opportunities for social interaction, emotional support, reduced loneliness, and improved emotional wellbeing (4–6). However, the benefits of digitalization are not equally accessible to all older adults. Differences in health status, educational attainment, and social support may contribute to substantial disparities in access to, use of, and opportunities to benefit from digital resources (7, 8), making digital participation an increasingly important issue from a health equity perspective.

Frailty is a geriatric syndrome reflecting multidimensional deficits across the physical, psychological, and social domains of later life (9). It is highly prevalent among adults aged 65 years and older and is associated with adverse outcomes, including falls, disability, long-term care dependency, and mortality (10). In recent years, increasing attention has been paid to the relationship between internet use and frailty in later life. On the one hand, previous studies have suggested that internet use may have a potentially protective role and have reported associations with lower levels of frailty, better cognitive function, and other favorable health outcomes. Potential pathways underlying these associations may include enhanced social connectedness, healthier behaviors, and greater access to health-related knowledge (11–15). On the other hand, frailty itself may also shape digital participation. Older adults with greater frailty may encounter multiple barriers when using smartphones and internet-based services, including visual, cognitive, mobility-related, and emotional limitations (16, 17). The relationship between internet use and frailty is therefore likely to be complex and context dependent.

However, existing research has predominantly relied on quantitative analyses of large-scale databases, typically operationalizing internet use in terms of use vs. non-use, frequency, or duration, while often treating older adults as a relatively homogeneous population (18, 19). Although such approaches are valuable for identifying overall statistical associations between internet use and health, they provide limited insight into why and how older adults use the internet, what barriers they encounter during use, and whether digital participation can be translated into health-related resources across different frailty statuses. Existing evidence therefore offers limited understanding of the heterogeneity of internet use and the contexts in which such differences arise. Qualitative inquiry is needed to provide a more contextualized understanding of the lived digital experiences of older adults with varying frailty statuses and of the behavioral and resource-related factors underlying these differences.

A health equity perspective provides an important analytical framework for understanding this heterogeneity. In the context of digital health, health equity concerns not only equitable access to conventional healthcare resources but also whether different populations are able to access, use, and benefit from digital resources according to their health needs (20–22). In this study, health equity refers to whether older adults with varying frailty statuses, socioeconomic backgrounds, and support conditions are able to access and use internet-based resources according to their differentiated health needs and have opportunities to translate digital participation into health-related resources and potential benefits. The three-level digital divide framework provides a further analytical lens for understanding this process. The framework conceptualizes digital inequality as occurring across three progressive levels. The first-level digital divide concerns access to digital technologies and the internet, that is, whether individuals are able to obtain and use relevant technologies. The second-level digital divide concerns differences in digital capability and patterns of use after access has been achieved. The third-level digital divide concerns whether digital participation can be translated into tangible economic, social, or health-related benefits (23, 24). In accordance with the aims of the present study, these three levels were specifically mapped onto: (1) the stability and autonomy of internet access; (2) digital capability and actual patterns of use; and (3) the capacity and opportunity to translate digital participation into health-related resources and potential benefits, including health information, digital healthcare services, and health-management resources. Importantly, because this study did not directly measure objective health outcomes resulting from internet use, the third level primarily reflects opportunities to translate digital participation into health-related resources and potential benefits rather than observed health effects.

User personas provide a suitable methodological approach for representing such multidimensional heterogeneity. Originating in the fields of human–computer interaction and service design, personas integrate information about the target population's sociodemographic attributes, behavioral patterns, needs, psychological characteristics, social relationships, and environmental contexts to construct representative user profiles that can inform differentiated product and service design (25). More recently, persona-based approaches have increasingly been applied in health services and health management for older adults to identify heterogeneous needs and support more tailored service development (26, 27). Compared with classification approaches based on isolated demographic or behavioral variables, qualitative user personas can integrate multiple dimensions, including health status, digital capability, patterns of use, social support, and contextual conditions, making them particularly suitable for representing the complexity and contextual variation of digital participation among older adults (25, 28).

## 2 Methods

### 2.1 Study design

This study employed a descriptive qualitative design. First, this approach is well suited to describing diverse experiences and behavioral patterns within heterogeneous populations, which aligns with the aim of exploring internet-use characteristics and health equity-related differences among older adults with varying frailty statuses. Second, unlike phenomenological research, which focuses on elucidating the essence of lived experiences, or grounded theory, which aims to generate new theory, descriptive qualitative research emphasizes a systematic, data-near description of patterns and their relevant contexts based closely on participants' original accounts. Third, this design allows the health equity perspective and the three-level digital divide framework to be applied as interpretive lenses after data collection and inductive analysis to understand differences across the identified personas. Data were collected through face-to-face semi-structured interviews conducted from October to December 2025. The study was reported in accordance with the Consolidated Criteria for Reporting Qualitative Research (COREQ) (29).

### 2.2 Participants and settings

The study was conducted from October to December 2025 in one community hospital and one tertiary hospital in Chongqing, China. Participants were recruited from the rehabilitation, cardiothoracic surgery, and gastroenterology departments. Based on factors previously reported to be associated with internet use among older adults (30–32), age, gender, urban–rural residence, educational attainment, and frailty status were considered the principal dimensions for maximum variation sampling. During recruitment, the research team continuously monitored sample composition and adjusted recruitment priorities accordingly. No strict numerical quotas were imposed, with the aim of increasing heterogeneity and coverage across these characteristics.

The inclusion criteria were as follows: (1) aged ≥60 years; (2) having experience with internet use, which in this study was defined as direct or assisted use of internet-enabled smartphones or other electronic devices and related applications; (3) having a basic awareness of their own health status and being able to clearly describe their internet-use experiences and related perceptions; and (4) voluntarily agreeing to participate and providing written informed consent. Individuals who were unable to complete the interview because of cognitive impairment or severe mental illness were excluded.

Sample size was determined by data saturation. The research team met regularly to review the interview process and emerging data, and recruitment was to cease when three consecutive interviews yielded no new information. Saturation was assessed across the dataset as a whole rather than separately within each frailty-status group; however, representation across different frailty statuses was continuously monitored during recruitment. After 21 participants had been interviewed and preliminary analysis had been conducted, no substantively new codes or categories were identified. Three additional confirmatory interviews were subsequently conducted, and no new information emerged. The research team therefore agreed that data saturation had been reached and stopped recruitment, resulting in a final sample of 24 older adults.

### 2.3 Frailty assessment

Frailty status was assessed using a Frailty Index (FI) based on the cumulative deficit model (33, 34). An FI typically comprises health deficits related to disease, disability, comorbidity, depressive symptoms, and other health conditions, with at least 30 deficits generally recommended (35). FI data were obtained through a structured assessment before the formal interview. The FI constructed in this study comprised 38 health deficits, including 14 chronic disease items, 3 self-rated health indicators, 7 physical-function items, 6 Activities of Daily Living (ADL) items, 6 Instrumental Activities of Daily Living (IADL) items, 1 depressive-symptom deficit, and 1 cognitive-function deficit. Detailed items and scoring procedures are provided in the Supplementary material 1. The FI was calculated by dividing the sum of accumulated deficit scores by the number of deficits assessed for each participant, yielding a score ranging from 0 to 1. Higher scores indicate a greater accumulation of health deficits. Based on previous research, participants were categorized as robust (FI ≤ 0.10), pre-frail (0.10 < FI < 0.25), or frail (FI ≥ 0.25) (36). FI scores and frailty categories were not used to stratify the initial qualitative coding or as predetermined criteria for persona grouping. Following the inductive analysis, frailty information was incorporated as a participant-level contextual attribute in individualized profiling and cross-case comparison.

### 2.4 Research team

The research team consisted of five researchers, all of whom had received training in qualitative research methods. Data collection was primarily conducted by two researchers, one holding a master's degree and the other a doctoral degree. Both researchers participated in all formal interviews. Before data collection began, the two interviewers standardized the interview procedures and probing strategies. The remaining three researchers comprised two experienced experts in geriatric nursing and one public health expert. They primarily provided methodological guidance throughout the study and participated in discussions and consensus building when disagreements arose during data analysis.

### 2.5 Data collection and interview guide

Data were collected through face-to-face semi-structured interviews. After the study purpose had been explained and voluntary consent obtained, baseline sociodemographic information was collected, including age, gender, marital status, educational attainment, place of residence, and living arrangements, to contextualize participants' patterns of internet use. The initial interview guide was developed on the basis of a literature review and the study research questions. Open-ended questions were used to encourage participants to describe their internet-use experiences, related behaviors, and contexts of use. Pilot interviews were subsequently conducted with three older adults who met the inclusion criteria. Based on participant feedback and interview observations, the two interviewers refined the wording, sequence, and probing strategies of the questions. The final interview guide was reviewed by experts in geriatric nursing and public health and is provided in the Supplementary material 2. Formal interviews were scheduled at times convenient for participants and were conducted in locations including community activity rooms and hospital education rooms. Each interview lasted approximately 30–60 m and was conducted by two researchers trained in qualitative methods. One researcher served as the primary interviewer and conducted probing, while the other assisted with observation, documentation of non-verbal information, and audio recording. Open-ended questions and appropriate probes were used until the researchers had obtained a sufficiently detailed understanding of participants' experiences and perspectives. All interviews were audio-recorded with participants' consent and transcribed verbatim.

### 2.6 Data analysis

#### 2.6.1 Establishing persona dimensions

Interview data were analyzed using inductive content analysis supported by *N Vivo* 12.0 (37). No *a priori* coding framework was imposed. The analysis proceeded as follows: (1) Transcription and familiarization: all interviews were transcribed verbatim and checked for accuracy. Two researchers repeatedly read the transcripts to become familiar with the data and develop an overall understanding of participants' internet-use experiences and related contexts. (2) Initial coding: meaning units related to internet-use behaviors, digital capability, barriers to use, social support, and health-related constraints were identified. Initial codes were generated while remaining as close as possible to participants' original accounts. (3) Code comparison and consensus: the two researchers compared the initial codes they had developed independently. Where discrepancies arose, the original transcripts were revisited and discussed until consensus was reached. If disagreement remained, a final decision was reached through research-team discussion. (4) Category and analytic-dimension development: codes with similar meanings and characteristics were continuously compared and grouped into lower-level subcategories, which were subsequently abstracted and inductively organized into higher-order descriptive categories. (5) Analytic verification: the research team repeatedly compared the emerging categories and analytic dimensions with the original interview data to assess their internal coherence and fit with the participants' accounts. Categories with overlapping meanings or unclear boundaries were revised and refined accordingly. This process ultimately generated four analytic dimensions: health status and self-management, digital participation foundation and capability, internet-use orientation and behavioral patterns, and constraints and enabling conditions. An illustrative example showing the progression from verbatim interview data through initial coding and category development to analytic dimensions, and its subsequent use in individualized profiling and cross-case comparison, is provided in Supplementary material 3.

#### 2.6.2 Extracting features and constructing user personas

Based on the four core analytic dimensions described above, the research team further extracted participant characteristics and constructed the user personas through the following steps: (1) Construction of individualized multidimensional profiles: key characteristics of each participant were extracted across the four core dimensions to construct an individualized multidimensional profile. The corresponding construction matrix is provided in the Supplementary material 4. (2) Participant validation of individualized profiles: the preliminary profiles were returned to the corresponding participants, who were asked to assess whether the profiles accurately reflected their internet-use experiences. Relevant descriptions were supplemented or revised according to participant feedback. (3) Cross-case comparison and preliminary grouping: the research team compared the 24 individualized profiles across dimensions and cases to identify recurring multidimensional configurations. The number of personas was not predetermined but emerged progressively through continuous comparison. Participants with similar overall configurations were assigned to the same preliminary persona group. The grouping matrix is provided in the Supplementary material 5. (4) Persona refinement and finalization: the research team repeatedly compared the original interview data, coding results, individualized profiles, and preliminary groupings. Personas with overlapping characteristics or unclear boundaries were merged, differentiated, or refined as appropriate. Cases with uncertain group membership were resolved through team discussion and consensus. Five final user personas were identified and named according to their distinguishing internet-use characteristics. The final personas were subsequently reviewed by the research team.

#### 2.6.3 Visualization and description of user personas

To facilitate intuitive presentation of the differentiated internet-use characteristics of older adults with varying frailty statuses, representative keywords were extracted from each persona. Keywords were selected by considering both their frequency of occurrence and contextual salience. WordArt was then used to generate word clouds as supplementary visual representations of the core characteristics of each persona.

Each persona was visually presented using a word cloud together with descriptive labels, while a summary table was used to compare multidimensional characteristics across personas. The word clouds served only as supplementary visual summaries and were not used in coding, persona classification, or the generation of analytic findings. After persona construction, the three-level digital divide framework was subsequently applied as an interpretive lens for cross-persona comparison.

### 2.7 Rigor

The rigor of the study was guided by the criteria of credibility, transferability, dependability, and confirmability (38). Triangulation across researcher analysis, participant validation, and expert review was used to strengthen the credibility and dependability of the findings. First, all researchers involved in interviewing and data analysis had received training in qualitative research methods. Interviews were conducted according to standardized procedures, and the researchers had no prior relationships with the participants. The research team maintained a neutral and non-leading stance throughout data collection and analysis. Second, participant validation was conducted in three stages. All participant validation was conducted individually rather than through group sessions. After transcription, the interview transcripts were returned to the corresponding participants to verify the accuracy of the content. After the preliminary individualized multidimensional profiles had been developed, these profiles were again returned to the respective participants to confirm whether they accurately reflected their internet-use experiences, and relevant descriptions were revised when necessary. All 24 participants completed these first two stages of validation, and none requested substantive revisions. After the five group-level personas had been finalized, one participant from each persona who was able to clearly articulate their experiences was invited to review the corresponding group-level persona. All five participants considered the persona descriptions to accurately reflect the internet-use characteristics of their respective groups and did not suggest substantive revisions. The personas were jointly reviewed by the research team, with the two geriatric nursing experts and one public health expert further evaluating the persona classification, defining characteristics, and naming. Transferability was supported through purposive sampling with a maximum variation strategy and by providing detailed descriptions of the study settings, participant characteristics, and research context, enabling readers to judge the applicability of the findings to similar settings. To enhance dependability and confirmability, two researchers independently participated in coding and comparison. Disagreements were resolved by revisiting the original data and reaching consensus through team discussion. An auditable analytic trail was maintained, including the interview guide, audio recordings and transcripts, coding records, individualized profiles, persona-construction matrices, and documentation of key analytic decisions. Throughout data collection and analysis, the research team also engaged in regular discussions of potential researcher assumptions and interpretive biases. Continual return to the original data and team reflexivity were used to minimize the influence of subjective researcher judgment on the findings.

## 3 Results

### 3.1 Participant characteristics

A total of 24 older adults participated in the study, including 14 men (58.33%) and 10 women (41.67%). Participants ranged in age from 63 to 83 years, with a mean age of 72.08 ± 5.78 years. Eight participants (33.33%) were aged 60–69 years, 14 (58.33%) were aged 70–79 years, and 2 (8.33%) were aged 80 years or older. Regarding educational attainment, 3 participants (12.50%) had no formal education, 4 (16.67%) had completed primary education, 13 (54.17%) had received secondary education (junior high school, senior high school, or technical secondary school), and 4 (16.67%) had received higher education (college diploma or above). Eighteen participants (75.00%) lived in urban areas and 6 (25.00%) in rural areas. Regarding living arrangements, 2 participants (8.33%) lived alone, 13 (54.17%) lived with a spouse, 5 (20.83%) lived with their children, and 4 (16.67%) lived with both a spouse and children. FI scores ranged from 0.06 to 0.56, with a mean of 0.22 ± 0.17. Based on the predefined FI thresholds, 7 participants (29.17%) were classified as robust, 10 (41.67%) as pre-frail, and 7 (29.17%) as frail. Participant characteristics are summarized in Table 1.

**Table 1:** Participant characteristics (*n* = 24).

Variable	Frequency (*n*)	Percentage (%)
Gender		
Male	14	58.33
Female	10	41.67
Age		
60–69	8	33.33
70–79	14	58.33
≥80	2	8.33
Education level		
No formal education	3	12.50
Primary education	4	16.67
Secondary education	13	54.17
Higher education	4	16.67
Residence		
Urban	18	75.00
Rural	6	25.00
Living arrangement		
Living alone	2	8.33
Living with spouse	13	54.17
Living with children	5	20.83
Living with spouse and children	4	16.67
Frailty status		
Robust (FI ≤ 0.10)	7	29.17
Pre-frail (0.10 < FI < 0.25)	10	41.67
Frail (FI ≥ 0.25)	7	29.17

### 3.2 User personas of internet use among older adults with varying frailty statuses

Following inductive content analysis and subsequent cross-case comparison, five internet-use personas were identified: Health-Empowered Integrators, Basic-Function Adapters, Entertainment-Oriented Users, Resource-Limited Users, and Health-Affected Users. The five personas represented distinct configurations across four core dimensions: health status and self-management, digital participation foundation and capability, internet-use orientation and behavioral patterns, and constraints and enabling conditions. Their sociodemographic characteristics and FI profiles are summarized in Table 2.

**Table 2:** User personas of internet use patterns among older adults with varying frailty statuses.

Characteristics	Health-empowered integrators	Basic-function adapters	Entertainment-oriented users	Resource-limited users	Health-affected users
Personas	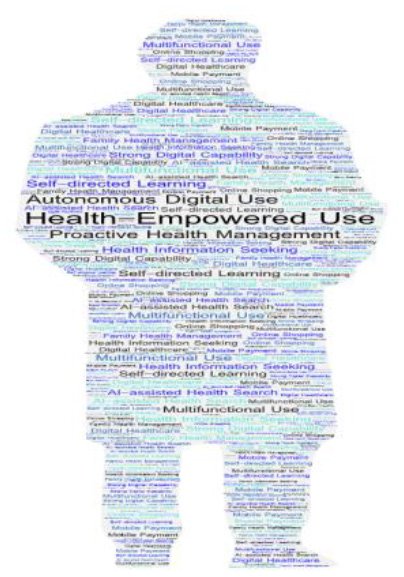	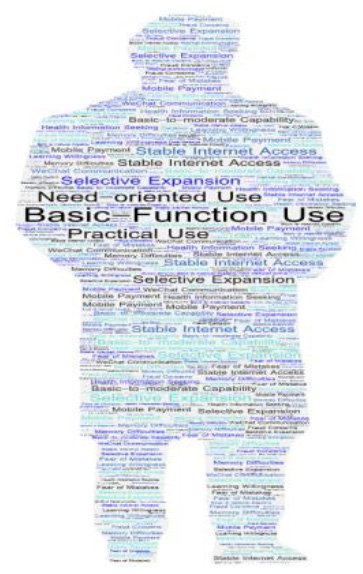	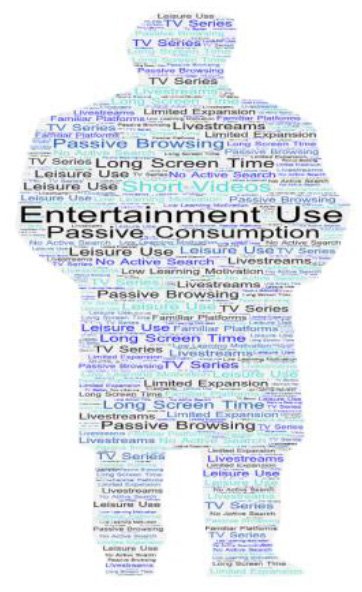	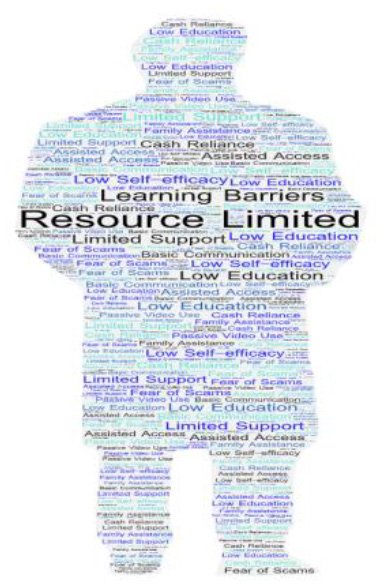	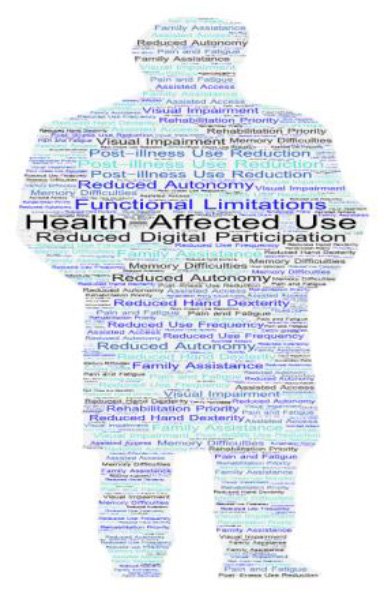
Participants	P9, P14, P24	P2, P4, P5, P12, P17, P18	P1, P21	P10, P11, P15, P16, P20, P22	P3, P6, P7, P8, P13, P19, P23
Basic characteristics					
Gender	3 males	6 males	1male, 1 female	2 males, 4females	2 males, 5 females
Mean age	71.7	74.7	66.0	71.2	72.6
Education level	Predominantly higher education	Predominantly secondary education	Primary and secondary education	Predominantly primary education or below	Secondary education
Residence	Urban	Predominantly urban	Both urban and rural areas	Predominantly rural	Urban
Mean FI	0.10	0.13	0.27	0.13	0.43
Health status and self-management	Proactive health information seeking; active self/family health management	Variable health status; selective, need-based health management	Limited active health management; health status varied	Limited digital health management; health not the primary constraint	Health- and functional limitations; reduced participation after illness
Digital participation foundation and capability	Stable access; proficient, autonomous, and extensible use	Stable access; basic-to-moderate capability; difficulty with complex functions	Stable access; limited active-search and service-use capability	Basic or assisted access; low autonomy and limited functional skills	Access retained but often reduced or assisted; health-constrained operation
Internet-use orientation and behavioral patterns	Proactive exploration; multifunctional and health-oriented use	Need-oriented use; selective expansion beyond basic functions	Entertainment-oriented; predominantly content-viewing use	Calls and passive video viewing; minimal digital-service use	Use narrowed after illness; familiar and essential functions prioritized
Constraints and enabling conditions	Few major barriers; strong self-learning capability with supplementary support	Memory, complexity, and risk concerns; support needed for unfamiliar tasks	Low motivation for functional expansion; family handles other digital tasks	Low education; device/data, learning-opportunity, and support constraints	Health-related functional limitations; reliance on family/caregiver assistance

#### 3.2.1 Health-empowered integrators

This persona was characterized by the integration of strong digital capability with proactive health-related use. Participants demonstrated high autonomy in learning and using digital technologies and were able to extend internet use beyond routine communication and entertainment to a broad range of everyday and health-related activities.

##### 3.2.1.1 Health status and self-management

Participants showed proactive health-information seeking and used digital resources to support their own or their family members' health management.

##### 3.2.1.2 Digital participation foundation and capability

Internet access was stable, and participants demonstrated proficient, autonomous, and extensible digital use, with strong self-learning capability.

##### 3.2.1.3 Internet-use orientation and behavioral patterns

Internet use was characterized by proactive exploration and multifunctional integration, including communication, mobile payment, online shopping, information seeking, **digital healthcare services**, and AI-assisted health-information seeking.

##### 3.2.1.4 Constraints and enabling conditions

Few major barriers were reported. Strong self-learning capability, previous learning or occupational experience, and supplementary support from family members or others facilitated continued digital participation.

“*I learned most of these things by myself. I later found an AI app and used it to search for hospitals in Chongqing that perform minimally invasive surgery for esophageal cancer. It listed several hospitals, and I used that information when choosing this hospital.”* (P9, male, 69 years, senior high school, FI = 0.06)

“*I can use mobile payment, online shopping, food delivery, and AI. I learned most of these functions on my own. Since my wife had a stroke, I have also used AI to ask specific questions about her rehabilitation and what we should pay attention to.”* (P24, male, 83 years, college diploma, FI = 0.10)

#### 3.2.2 Basic-function adapters

This persona represented an intermediate pattern in which participants had established stable basic-to-moderate digital capability but encountered difficulties extending their use to more complex or unfamiliar functions. Their internet use was generally practical and need-oriented, with selective rather than continuous expansion.

##### 3.2.2.1 Health status and self-management

Health status varied across participants. Health-related internet use was generally selective and need-driven rather than consistently integrated into self-management.

##### 3.2.2.2 Digital participation foundation and capability

Participants generally had stable internet access and could perform basic to moderately complex functions, but difficulties emerged in skill retention, complex procedures, or unfamiliar digital tasks.

##### 3.2.2.3 Internet-use orientation and behavioral patterns

Internet use was predominantly need-oriented, with selective expansion beyond familiar functions according to practical needs.

##### 3.2.2.4 Constraints and enabling conditions

Memory difficulties, procedural complexity, concerns about fraud or operational errors, and inconsistent support for unfamiliar tasks were recurrent barriers.

“*I even bought a book to learn how to use a smartphone and wrote the steps down in a notebook. I more or less learned them, but I still find some functions difficult because I forget the steps.”* (P4, male, 75 years, technical secondary school, FI = 0.07)

“*If something can be handled face to face, I would rather do it offline. Online, I worry that one wrong tap will make things more complicated.”* (P2, male, 67 years, college diploma, FI = 0.11)

“*I can usually figure things out by myself, and I often search for rehabilitation and health information. But I avoid functions involving money, especially transfers, because I am afraid of making a mistake.”* (P5, male, 78 years, junior high school, FI = 0.21)

#### 3.2.3 Entertainment-oriented users

This persona was not characterized primarily by restricted internet access, but by a relatively concentrated pattern of use centered on entertainment and content viewing. Participants were able to use familiar digital platforms consistently, yet rarely extended their use to activities such as information seeking, online services, or health management. For this group, the internet functioned mainly as a source of leisure and everyday enrichment.

##### 3.2.3.1 Health status and self-management

Health status varied between participants, but active use of digital resources for health management was limited.

##### 3.2.3.2 Digital participation foundation and capability

Participants had relatively stable access and could use familiar functions, but active searching and service-oriented digital capability were limited.

##### 3.2.3.3 Internet-use orientation and behavioral patterns

Internet use was strongly entertainment-oriented and centered on passive content consumption, particularly short videos, television series, livestreams, or similar content.

##### 3.2.3.4 Constraints and enabling conditions

Low perceived need for functional expansion and reliance on family members for other digital tasks limited broader digital participation.

“*I mainly watch TV series, sometimes play cards, and scroll through Douyin. I can spend 4 or 5 h on it, especially at night when I cannot sleep.”* (P1, male, 69 years, primary education, FI = 0.38)

“*I use it for at least 5 or 6 h a day. I mostly scroll through videos—whatever comes up. I do not know how to search; I mainly watch short dramas and livestreams.”* (P21, female, 63 years, junior high school, FI = 0.15)

#### 3.2.4 Resource-limited users

This persona was characterized primarily by limited access to the resources and support needed for sustained digital participation. Although participants had some experience with internet use, limited educational and digital-learning resources, insufficient ongoing support, and low confidence made it difficult to develop stable autonomous use. Their internet activities therefore tended to remain confined to a small number of familiar functions and frequently depended on family assistance.

##### 3.2.4.1 Health status and self-management

Participants generally demonstrated limited proactive health management and relatively low self-efficacy, particularly regarding their perceived ability to learn and use unfamiliar digital technologies.

##### 3.2.4.2 Digital participation foundation and capability

Internet access and routine operation frequently depended on assistance from family members. Autonomous digital skills were limited, and some functions required preconfiguration or direct assistance from others.

##### 3.2.4.3 Internet-use orientation and behavioral patterns

Internet use was largely confined to basic communication and simple entertainment functions and was frequently assistance-dependent. Independent use of functions such as online shopping and mobile services was uncommon.

##### 3.2.4.4 Constraints and enabling conditions

Limited educational resources, insufficient opportunities for digital learning, and weak intergenerational support were prominent constraints. Unlike the Health-Affected Users, the defining limitations in this group were primarily related to available resources and support rather than health-related functional impairment.

“*I really cannot use most of the functions. I can only make and answer phone calls. They have tried to teach me, but I still do not understand and cannot remember the steps.”* (P10, female, 63 years, no formal education, FI = 0.18)

“*I only use WeChat for calls and watch whatever videos come up. I do not know how to use payment or messaging. I do not really want to learn—I feel I do not have much education, and my son has not taught me these things.”* (P20, male, 72 years, primary education, FI = 0.13)

“*I do not really know how to use it; my daughter sets up the videos for me. I do not use WeChat, and I am afraid of being scammed. I also have to care for my husband and work, so I only have a little time at night to look at my phone.”* (P22, female, 71 years, no formal education, FI = 0.08)

#### 3.2.5 Health-affected users

This persona was characterized by health- or function-related limitations that constrained the scope, frequency, or autonomy of digital participation. Importantly, several participants had previously been able to perform a broader range of digital activities, but illness, sensory impairment, motor limitations, or cognitive changes subsequently narrowed their use.

##### 3.2.5.1 Health status and self-management

Physical, sensory, or cognitive limitations were prominent. For several participants, illness or functional decline directly altered their willingness or ability to engage with digital technologies.

##### 3.2.5.2 Digital participation foundation and capability

Internet access was generally retained, but the autonomy and ease of use were often reduced. Some participants increasingly relied on family members or caregivers for device settings or complex operations.

##### 3.2.5.3 Internet-use orientation and behavioral patterns

Digital use often narrowed after illness or functional decline, with familiar and essential activities prioritized over learning new functions or using more complex services.

##### 3.2.5.4 Constraints and enabling conditions

Health-related functional limitations constituted the dominant barriers, while family or caregiver assistance helped maintain basic or adapted digital participation.

“*Since I became ill, I hardly use my phone. My hands and feet are not as flexible as before, and tapping the screen has become difficult. I also contact people much less than I used to.”* (P8, male, 78 years, junior high school, FI = 0.56)

“*I rarely use my phone except for making calls because I have macular degeneration and cannot see clearly. I can still use WeChat Pay, but my daughter enlarged the text on my phone for me.”* (P19, male, 75 years, technical secondary school, FI = 0.13)

“*Before I became ill, I was willing to learn new functions. Now I do not want to learn them—I just want to recover and be able to take care of myself again. I also contact far fewer people than before.”* (P23, female, 78 years, technical secondary school, FI = 0.52)

### 3.3 Differences across user personas through the lens of the three-level digital divide

After the five personas had been identified, the three-level digital divide framework was further applied as an interpretive lens to compare patterns of digital participation across the personas. In the present study, the three levels corresponded to internet access; digital capability and actual patterns of use; and the capacity and opportunity to translate digital participation into health-related resources and potential benefits. The specific mapping is presented in Table 3.

**Table 3:** Mapping of the five user personas onto the three-level digital divide framework.

User personas	First-level digital divide	Second-level digital divide	Third-level digital divide	Key health equity-related conditions
Health-empowered integrators	Stable and autonomous access	High autonomy; multifunctional use	Greater capacity and opportunity to translate digital participation into health-related resources and potential benefits	Strong digital capability and supportive resources
Basic-function adapters	Stable access	Basic use; limited capability for complex functions	Limited capacity to translate digital participation into health-related resources and potential benefits	Difficulties retaining skills, risk concerns, and insufficient ongoing support
Entertainment-oriented users	Stable access	Narrow scope of use; predominantly entertainment-oriented	Limited use of health-related resources and limited opportunities for further translation into potential benefits	Concentrated use orientation; limited perceived need for functional expansion
Resource-limited users	Access often dependent on assistance	Limited autonomous use	Limited opportunities to use digital health resources	Insufficient education, learning resources, and intergenerational support
Health-affected users	Intermittent or assisted access	Constrained by health-related functional limitations	Limited opportunities to use health-related digital resources	Physical, sensory, and cognitive functional limitations

## 4 Discussion

Based on the internet-use experiences of older adults with varying frailty statuses, this study identified five user personas. The findings indicate that digital participation among older adults cannot be adequately characterized simply by whether they use the internet or how frequently they use it. Frailty status represents an important health-related context for understanding these differences, but it is not the sole determinant. Rather, different configurations of digital capability, use orientation, social support, and resource conditions jointly shape how older adults engage with the internet and their opportunities to access and use digital health resources.

### 4.1 Differences across the five user personas and implications for health equity

#### 4.1.1 Health-Empowered Integrators

Health-Empowered Integrators demonstrated relatively strong digital capability and experienced fewer frailty-related constraints, enabling them to integrate internet use into both health management and everyday life. Across the three levels of the digital divide, these participants showed relative advantages: they had stable and autonomous internet access, engaged in multifunctional digital use, and were able to extend their digital participation to health information, digital healthcare services, and health-management resources. This pattern may reflect the combined influence of educational attainment, self-efficacy, and supportive resources. Previous studies have shown that higher educational attainment facilitates the comprehension of digital information and the acquisition of new skills, while stronger digital self-efficacy is associated with more active internet use among older adults (39, 40). Family support and continued digital practice may further contribute to the maintenance of digital capability and the expansion of digital functions (41, 42). Our findings therefore suggest that stronger capabilities and resource bases may make it easier for older adults to broaden their digital participation and access a wider range of health-related resources. At the same time, this pattern may reflect a potential digital Matthew effect, whereby groups with existing advantages may be better positioned to consolidate those advantages through digital technologies, potentially widening health-related disparities if inequities in access to resources remain unaddressed (43, 44). Accordingly, support for this group may shift from basic skills training toward digital health literacy, health-information verification, and the safe use of emerging digital tools. Where appropriate and voluntary, these older adults may also serve as peer educators or digital health supporters for others.

#### 4.1.2 Basic-function adapters

Basic-function adapters represented a relatively large intermediate group. They were able to access the internet consistently and complete basic activities such as communication and mobile payment, but experienced difficulties learning more complex functions, retaining digital skills, and independently managing digital tasks. Their primary disadvantages therefore did not lie in the first-level digital divide, but were concentrated at the second level and subsequently affected their ability to access health-related resources at the third level. Experiences such as “learning but easily forgetting,” concerns about online fraud, and lack of confidence when dealing with complex functions repeatedly emerged among this group. Previous research has suggested that age-related changes in hippocampal functioning and cognitive decline may contribute to difficulties retaining digital skills (45, 46). Compared with younger adults, older adults may also have greater concerns about online fraud and information leakage (46), while digital anxiety may reduce their willingness to engage with digital technologies (47). However, our findings further suggest that a restricted range of internet use does not necessarily indicate a lack of willingness to learn. When operating procedures are complex, functions are frequently updated, and the perceived consequences of errors are high, remaining within familiar digital activities may also represent an adaptive strategy to reduce cognitive burden and perceived risk. The health equity risks faced by this group are therefore relatively inconspicuous: although they appear to have achieved digital access, limitations in capability may still hinder their effective use of digital health resources. Support should therefore move beyond one-off instruction toward skill reinforcement, repeated practice, and context-based training, while also strengthening fraud awareness and privacy protection.

#### 4.1.3 Entertainment-oriented users

Entertainment-oriented users had relatively stable internet access, but their digital activities were concentrated primarily on entertainment, such as short videos and television series. Their main disadvantage was therefore evident in the second-level digital divide, where the range and orientation of internet use were relatively narrow, which in turn limited access to third-level resources such as health information, public services, and health management. In this study, some participants regarded entertainment-oriented internet use as a way to occupy free time and enrich everyday life, while also reporting relatively limited offline social participation. This pattern suggests that entertainment use may serve a compensatory role when social roles or offline activities become restricted and should not simply be interpreted as “negative” or “ineffective” internet use. From a social capital perspective, previous studies have shown that social participation among older adults is associated with a lower risk of depression (48, 49), better cognitive function (50, 51), and better physical health (52, 53). However, digital access may acquire greater health-related value only when it extends to social interaction, information acquisition, and resource connection (54, 55). In the present study, digital activity among Entertainment-Oriented Users remained largely centered on content consumption and less frequently extended to active interaction, information seeking, or the use of health-related resources. Previous research has also suggested that excessive internet use may be associated with greater risks of depression and anxiety (56), while prolonged sedentary behavior is associated with obesity, cardiovascular disease, and diabetes (57). Accordingly, interventions for this group could integrate credible health information into familiar entertainment contexts, provide low-threshold opportunities for online social interaction, introduce age-friendly behavioral guidance, and strengthen intergenerational and community-based offline engagement to reduce excessive digital dependence and related health risks.

#### 4.1.4 Resource-limited users

From the perspective of the three-level digital divide, Resource-Limited Users experienced constraints across internet access, digital capability, and the translation of digital participation into health-related resources. At the first level, their disadvantage did not necessarily involve complete lack of internet access; rather, access was often dependent on family assistance. Limited educational foundations, insufficient opportunities for digital learning, and inadequate sustained support made it difficult for such assisted access to develop into stable autonomous digital use, which in turn restricted opportunities to use digital health resources. Within our sample, participants in this group more often had lower levels of education and rural backgrounds, and women accounted for a relatively larger proportion. Previous studies have shown that educational attainment, urban–rural differences in digital infrastructure, and socioeconomic resources are associated with digital technology adoption among older adults (58–60). Educational background may influence the ability to learn new technologies and process information, while urban–rural resource differences may affect access to digital training and sustained support. In addition, traditional gender roles and the cumulative distribution of family responsibilities may mean that older women undertake greater caregiving and domestic workloads, potentially compounding gender-related health disparities (61, 62). Addressing the disadvantages faced by this group therefore requires more than technical training alone. Structural interventions are also needed, including targeted digital-literacy programs for older adults with lower incomes or educational attainment, strengthened rural digital infrastructure, and coordinated multisectoral support to address intersecting forms of disadvantage.

#### 4.1.5 Health-affected users

Health-affected users experienced the most prominent health-related constraints among the five personas. Physical discomfort, visual impairment, reduced hand dexterity, and cognitive changes substantially narrowed both the frequency and scope of their internet use. Across the three levels of the digital divide, these health-related limitations affected the continuity and autonomy of internet access, increased the difficulty of digital operations, and ultimately reduced opportunities to use health information, digital healthcare services, and other health-related resources. Compared with the other personas, the most salient health equity concern for this group was a potential mismatch between health needs and access to digital services. As health status declines, older adults may have increasing needs for appointment booking, consultation, rehabilitation guidance, and health information, while physical, sensory, and cognitive limitations may simultaneously make these digital services more difficult to use. Previous research has also shown that illness-related psychological and social changes, including reduced self-efficacy, feelings of helplessness, and concerns about burdening others, may reduce willingness to accept digital assistance or participate in social activities (22). Moreover, digital products and services that do not adequately account for age-related changes in vision, fine motor function, and cognition may create additional barriers to use (46). Support for Health-Affected Users should therefore integrate digital inclusion with healthcare and functional support. This may include more accessible digital design, home-based digital assistance, and support in obtaining necessary health information and social resources.

### 4.2 Contextual relationship between frailty and digital participation

Comparison across the five personas further showed that frailty severity did not correspond directly to patterns of internet use. Digital participation among Health-Affected Users was primarily constrained by physical, sensory, and cognitive limitations, whereas Resource-Limited Users could experience limited autonomous digital use despite relatively lower frailty levels because of disadvantages in educational background, opportunities for digital learning, and sustained support. At the same time, some older adults with health-related limitations were still able to maintain basic digital activities by relying on previously acquired skills and family support. These findings suggest that frailty is an important health-related condition shaping digital participation, but its influence needs to be understood within the broader context of digital capability, social support, and available resources. In other words, similar health conditions do not necessarily correspond to similar patterns of digital participation.

These findings also provide a more nuanced understanding of the relationship between internet use and frailty. Previous quantitative studies have commonly reported associations between internet use and lower frailty levels or better health status (18, 19, 63–65). The experiences reported in the present study suggest another possible pathway: changes in health and functional status may themselves influence digital participation. Some participants reduced their previous internet activities after becoming ill or experiencing functional decline, suggesting that observed associations between internet use and health may reflect both the relationship of digital participation with health and the influence of health status on the ability to participate digitally. Because this was a cross-sectional qualitative study, no causal direction can be established. Nevertheless, these findings suggest that future research should move beyond conceptualizing internet use solely as a unidirectional influence on health and should use longitudinal designs to further examine possible bidirectional relationships while incorporating contextual factors such as digital capability, patterns of use, and support resources.

### 4.3 Limitations and future directions

This study has several limitations. First, all participants were recruited from healthcare settings, which may have resulted in greater representation of older adults who had more frequent contact with healthcare services, more prominent health problems, or greater familiarity with digital health services. The transferability of the findings to the general community-dwelling older population should therefore be considered with caution. Future studies could extend recruitment to different geographical regions, rural communities, and broader community-based populations. Second, the study included only older adults with previous experience of internet use and therefore did not capture those who had never used the internet. Never-users may face more fundamental barriers related to devices, connectivity, financial resources, and social support. Future research could compare the digital participation experiences and needs of never-users, assisted users, and autonomous users. Third, the cross-sectional design means that the five personas represent configurations observed at a particular point in time. The study therefore cannot establish the causal direction of the relationship between internet use and frailty or capture potential transitions between personas as health status, digital capability, and support resources change. Longitudinal qualitative or mixed-methods studies are needed to examine these dynamic transitions. Finally, the study relied primarily on self-reported interview data. In addition, the third-level digital divide in this study reflects participants' capacity and opportunity to translate digital participation into health-related resources and potential benefits, rather than objectively measured health outcomes. Accordingly, the health equity mechanisms discussed in this study should be understood as interpretations derived from qualitative data and existing theory rather than as verified causal mechanisms. Future studies could incorporate objective digital-use records, behavioral observation, and health indicators to further examine the personas and their health-related implications.

## 5 Conclusion

This study employed a descriptive qualitative design to develop five internet-use personas among older adults with varying frailty statuses. The findings showed that internet use among older adults is heterogeneous. Frailty represents an important health-related context influencing digital participation, but it does not determine internet-use patterns in a uniform manner. Rather, these patterns are jointly shaped by health-related functional limitations, digital capability, use orientation, social support, and resource conditions, which may further influence older adults' opportunities to access and use health-related digital resources. Therefore, differentiated support should be provided according to the characteristics and needs of different user personas to promote more equitable digital participation among older adults.

## Data Availability

The original contributions presented in the study are included in the article/Supplementary material, further inquiries can be directed to the corresponding author/s.
