# Supplementary material for: Internet use patterns among older adults with different frailty statuses: a qualitative user persona study from a health equity perspective
Source: Front Public Health. 2026 Oct 8;14:1912591. doi: 10.3389/fpubh.2026.1912591 (PMC13686913; doi:10.3389/fpubh.2026.1912591)
Supplement: Supplementary file 1 [file Table_1.docx]

## **Supplementary Material 1**

**Frailty Index Survey**

| Chronic Diseases | Questions | Answers | Values |
| --- | --- | --- | --- |
|  | Do you have any of the following diseases? | ①Hypertension | **yes=1; no=0;** |
|  |  | ②Hyperlipidemia |  |
|  |  | ③Diabetes or Elevated Blood Glucose |  |
|  |  | ④Malignant Tumors such as Cancer |  |
|  |  | ⑤Chronic Lung Diseases (e.g., Chronic Bronchitis, Emphysema, Cor Pulmonale; excluding tumors or cancer)） |  |
|  |  | ⑥Liver Diseases (excluding Fatty Liver, Tumors or Cancer) |  |
|  |  | ⑦Heart Diseases (e.g., Myocardial Infarction, Coronary Heart Disease, Angina Pectoris, Congestive Heart Failure, and other heart diseases) |  |
|  |  | ⑧Stroke (including Cerebral Infarction and Cerebral Hemorrhage) |  |
|  |  | ⑨Kidney Diseases (excluding Tumors or Cancer) |  |
|  |  | ⑩Gastric or Digestive System Diseases (excluding Tumors or Cancer) |  |
|  |  | ⑪Emotional and Mental Problems |  |
|  |  | ⑫Memory-related Diseases (e.g., Alzheimer’s Disease, Brain Atrophy, Parkinson’s Disease)） |  |
|  |  | ⑬Arthritis or Rheumatism |  |
|  |  | ⑭Asthma |  |

| Self-rated Health Status | Questions | Answers | Remarks |
| --- | --- | --- | --- |
|  | 1.How is your near vision? For example, can you read newspapers with glasses on? (Interviewer note: For regular glasses wearers, ask about vision with glasses on) | ①Excellent=0  ②Very Good=0.25  ③Good=0.5  ④Fair=0.75  ⑤Poor=1 |  |
|  | 2.How is your hearing? (If you wear a hearing aid regularly, how is your hearing with the hearing aid on? If you don’t wear a hearing aid regularly, how is your hearing without it?) | ①Excellent=0  ②Very Good=0.25  ③Good=0.5  ④Fair=0.75  ⑤Poor=1 |  |
|  | 3.How would you rate your overall health status? | ①Excellent=0  ②Very Good=0.25  ③Good=0.5  ④Fair=0.75  ⑤Poor=1 |  |

| Mobility Assessment | Questions | Answers | Remarks |
| --- | --- | --- | --- |
|  | 1.Do you have difficulty walking 1 kilometer? | ①No Difficulty=0  ②Have Difficulty but Can Still Complete=0.33  ③Have Difficulty and Need Assistance=0.67  ④Unable to Complete=1 |  |
|  | 2.Do you have difficulty walking 100 meters? | ①No Difficulty=0  ②Have Difficulty but Can Still Complete=0.33  ③Have Difficulty and Need Assistance=0.67  ④Unable to Complete=1 |  |
|  | 3.Do you have difficulty standing up from a chair after sitting for a long time? | ①No Difficulty=0  ②Have Difficulty but Can Still Complete=0.33  ③Have Difficulty and Need Assistance=0.67  ④Unable to Complete=1 |  |
|  | 4.Do you have difficulty bending over, kneeling down, or squatting? | ①No Difficulty=0  ②Have Difficulty but Can Still Complete=0.33  ③Have Difficulty and Need Assistance=0.67  ④Unable to Complete=1 |  |
|  | 5.Do you have difficulty raising your arms above your shoulders? (Considered no difficulty only if both arms can do it without trouble; otherwise, it is considered difficult) | ①No Difficulty=0  ②Have Difficulty but Can Still Complete=0.33  ③Have Difficulty and Need Assistance=0.67  ④Unable to Complete=1 |  |
|  | 6.Do you have difficulty lifting an object weighing 5 kilograms (10 jin)? | ①No Difficulty=0  ②Have Difficulty but Can Still Complete=0.33  ③Have Difficulty and Need Assistance=0.67  ④Unable to Complete=1 |  |
|  | 7.Do you have difficulty picking up a small coin from a table? | ①No Difficulty=0  ②Have Difficulty but Can Still Complete=0.33  ③Have Difficulty and Need Assistance=0.67  ④Unable to Complete=1 |  |

|  | Questions | Answers | Remarks |
| --- | --- | --- | --- |
| ADL | 1.Do you have difficulty dressing yourself due to health or memory problems? (Dressing includes taking clothes from the wardrobe, putting them on, buttoning up, and fastening the belt) | ①No Difficulty=0  ②Have Difficulty but Can Still Complete=0.33  ③Have Difficulty and Need Assistance=0.67  ④Unable to Complete=1 |  |
|  | 2.Do you have difficulty taking a bath due to health or memory problems? | ①No Difficulty=0  ②Have Difficulty but Can Still Complete=0.33  ③Have Difficulty and Need Assistance=0.67  ④Unable to Complete=1 |  |
|  | 3.Do you have difficulty feeding yourself (e.g., picking up food with chopsticks or a spoon) due to health or memory problems? (Definition: Feeding yourself refers to eating after meals are prepared) | ①No Difficulty=0  ②Have Difficulty but Can Still Complete=0.33  ③Have Difficulty and Need Assistance=0.67  ④Unable to Complete=1 |  |
|  | 4.Do you have difficulty getting up from bed? | ①No Difficulty=0  ②Have Difficulty but Can Still Complete=0.33  ③Have Difficulty and Need Assistance=0.67  ④Unable to Complete=1 |  |
|  | 5.Do you have difficulty using the toilet (including squatting down and standing up) due to health or memory problems? | ①No Difficulty=0  ②Have Difficulty but Can Still Complete=0.33  ③Have Difficulty and Need Assistance=0.67  ④Unable to Complete=1 |  |
|  | 6.Do you have difficulty controlling urination and defecation due to health or memory problems? (Considered able to control if you can use a catheter or urine bag independently) | ①No Difficulty=0  ②Have Difficulty but Can Still Complete=0.33  ③Have Difficulty and Need Assistance=0.67  ④Unable to Complete=1 |  |
| IADL | 7.Do you have difficulty doing housework due to health or memory problems? (Definition: Housework refers to cleaning the house, washing dishes, making the bed, and arranging the room) | ①No Difficulty=0  ②Have Difficulty but Can Still Complete=0.33  ③Have Difficulty and Need Assistance=0.67  ④Unable to Complete=1 |  |
|  | 8.Do you have difficulty cooking meals due to health or memory problems? (Definition: Cooking refers to preparing ingredients, cooking dishes, and serving them on the table) | ①No Difficulty=0  ②Have Difficulty but Can Still Complete=0.33  ③Have Difficulty and Need Assistance=0.67  ④Unable to Complete=1 |  |
|  | 9.Do you have difficulty going to the store to buy groceries due to health or memory problems? (Buying groceries here refers to deciding what to buy and paying for it) | ①No Difficulty=0  ②Have Difficulty but Can Still Complete=0.33  ③Have Difficulty and Need Assistance=0.67  ④Unable to Complete=1 |  |
|  | 10.Do you have difficulty making phone calls due to health or memory problems? | ①No Difficulty=0  ②Have Difficulty but Can Still Complete=0.33  ③Have Difficulty and Need Assistance=0.67  ④Unable to Complete=1 |  |
|  | 11.Do you have difficulty taking medication by yourself due to health or memory problems? (Taking medication refers to remembering when to take it and the dosage) | ①No Difficulty=0  ②Have Difficulty but Can Still Complete=0.33  ③Have Difficulty and Need Assistance=0.67  ④Unable to Complete=1 |  |
|  | 12.Do you have difficulty managing money (e.g., paying bills, keeping track of expenses, managing finances) due to health or memory problems? | ①No Difficulty=0  ②Have Difficulty but Can Still Complete=0.33  ③Have Difficulty and Need Assistance=0.67  ④Unable to Complete=1 |  |

| CESD-10 Depression Scale | Item Content | Rarely or None (<1 day) | Somewhat (1-2 days) | Occasionally or Half the Time (3-4 days) | Most of the Time (5-7 days) |
| --- | --- | --- | --- | --- | --- |
|  | Values | 0 | 1 | 2 | 3 |
|  | 1.I was bothered by things that usually don't bother me |  |  |  |  |
|  | 2.I had trouble keeping my mind on what I was doin |  |  |  |  |
|  | 3.I felt depressed |  |  |  |  |
|  | 4.I felt that everything I did was an effort |  |  |  |  |
|  | 5. I felt hopeful about the future |  |  |  |  |
|  | 6.I felt fearful |  |  |  |  |
|  | 7.My sleep was restless |  |  |  |  |
|  | 8.I felt happy |  |  |  |  |
|  | 9.I felt lonely |  |  |  |  |
|  | 10.I felt that I could not go on |  |  |  |  |

Respondents selected the options that best matched their actual conditions based on their feelings over the past week. Each item had four response options scored from 0 to 3 points (Items 5 and 8 were reverse‑scored). After completing all 10 items, the total score was obtained by summing all item scores. Following Andresen’s recommendation, a uniform standard cut‑off value of ≥10 was adopted for the total score of depressive symptoms. Accordingly, CES‑D‑10 total score ≥10 was coded as 1, and ＜10 was coded as 0.

| Cognitive Tests |  | Item | Questions | Answers |
| --- | --- | --- | --- | --- |
|  | Temporal Orientation Test | Year Orientation | “What year is it now?” | Correct=1；  Incorrect=0 |
|  |  | Month Orientation | "What month is it now?" | Correct=1；  Incorrect=0 |
|  |  | Date Orientation | "What is the date today?" | Correct=1；  Incorrect=0 |
|  |  | Day Orientation | "What day of the week is it today?" | Correct=1；  Incorrect=0 |
|  |  | Season Orientation | "What season is it now?" | Correct=1；  Incorrect=0 |
|  | Serial 7s Test | Please calculate 100 minus 7, then subtract 7 from the result, and keep doing this until I say "stop". | "What is 100 minus 7?" | Correct=1；  Incorrect=0 |
|  |  |  | "What is the result minus 7 again?" | Correct=1；  Incorrect=0 |
|  |  |  | "What is the result minus 7 again?" | Correct=1；  Incorrect=0 |
|  |  |  | "What is the result minus 7 again?" | Correct=1；  Incorrect=0 |
|  |  |  | "What is the result minus 7 again?" | Correct=1；  Incorrect=0 |

| Cognitive Tests | Word Recall Test | I will show you ten words. Please read them aloud. Then I will ask you to recall these ten words. (Present the 10 words horizontally one by one, 2 seconds per word. After presenting all words, ask the respondent to recall and say all the words they can remember immediately; tick the correctly recalled words in the list) | Immediate Recall Cream, Arm, Seaside, Letter, Queen, Wooden House, Shoulder Pole, Ticket, Grass, Engine | Score: Number of correctly recalled words ÷ 10 (continuous value from 0 to 1) |
| --- | --- | --- | --- | --- |
|  |  |  | Delayed Recall (after 4-5 minutes interval) Cream, Arm, Seaside, Letter, Queen, Wooden House, Shoulder Pole, Ticket, Grass, Engine | Score: Number of correctly recalled words ÷ 10 (continuous value from 0 to 1) |

The cognitive test consisted of four components: immediate and delayed word recall, date naming, and serial‑7 subtraction test. One point was awarded for each correct answer. The cognitive score was calculated using the following formula: (Total score − (immediate‑plus‑delayed word recall + date naming + serial‑7 subtraction)) / Total score. The total score refers to the theoretical maximum score for all tests. Accordingly, a higher cognitive score indicates poorer cognitive function.

**Supplementary Material 2**

**Interview Guide**

1.Which internet-enabled devices do you use? What was the initial reason for you to start using a mobile phone?

2.What do you mainly use the internet for? What is your favorite function or application?

3.What time of day do you typically use the internet, how long do you use it daily, and why do you prefer that particular time?

4.Does prolonged screen time cause any physical discomfort? Does using your phone reduce your offline activities? Have you ever used your phone to improve your health?

5.Does using a mobile phone affect your memory or mental agility? Do you enjoy the mental engagement while using the internet?

6.How do you feel when you successfully use your phone to complete daily tasks? Do you ever feel anxious due to operational difficulties or exposure to harmful online information?

7.Has online communication deepened your relationships with family and friends? How does making friends online differ from doing so offline?

8. After learning new phone functions, have you noticed any changes in your ability to handle daily life or in your self-confidence? How do you usually solve problems with your phone, and why?

9.Do you actively search for information online about topics that interest you? When learning a new function, is your attitude more resistant or curious?

10.Has using a mobile phone changed your daily routine or activity schedule? Are you able to control the amount of time you spend on it?

11.Are your children or grandchildren patient when teaching you how to use the phone? Do you have a peer support group for learning together?

12.Overall, has the internet made your life in older age richer or more troublesome? Does it make you feel more energetic or more fatigued?

13.Has the frequency or way you use your phone changed before and after illness? If so, why?

14.What are the main difficulties you encounter when using internet devices? What kind of support do you wish to receive?

**Supplementary Material 3**

**Illustrative Example of the Inductive Content Analysis Process**

| **Verbatim Interview Data / Meaning Unit** | **Initial Codes** | **Subcategory** | **Higher-Order Descriptive Category** | **Analytic Dimension** |
| --- | --- | --- | --- | --- |
| “I only use WeChat for calls and watch whatever videos come up. I do not know how to use payment or messaging.” (P20) | Use limited to basic calling functions; inability to use mobile payment; inability to send messages; limited functional digital skills | Limited mastery of basic digital functions | Limited autonomy and functional digital capability | **Digital Participation Foundation and Capability** |
| “I watch whatever videos come up.” (P20) | Passive viewing of recommended content; limited active content selection; lack of active searching | Passive content use | Narrow use pattern centered on basic communication and passive content viewing | **Internet-Use Orientation and Behavioral Patterns** |
| “I feel I do not have much education, and these things are difficult for me to learn.” (P20) | Low educational foundation; difficulty learning digital functions; low confidence in digital learning | Limited foundation for digital learning | Educational and learning-resource constraints | **Constraints and Enabling Conditions** |
| “My son has not specifically taught me how to use these functions.” (P20) | Lack of sustained family instruction; insufficient support for digital learning | Insufficient ongoing learning support | Limited family and learning-support resources | **Constraints and Enabling Conditions** |

**Supplementary Material 4**

**Individualized Multidimensional Profiles of the 24 Participants**

| **Participant** | **FI** | **Health status and self-management** | **Digital participation foundation and capability** | **Internet-use orientation and behavioral patterns** | **Constraints and enabling conditions** |
| --- | --- | --- | --- | --- | --- |
| **P1** | 0.38 | Higher frailty level; internet and television use decreased substantially during periods of physical discomfort. Little active use of digital resources for health management was evident. | Able to perform basic calling and simple WeChat voice/video functions; unable to conduct active searches or mobile payments. Initial digital skills were mainly taught by his son. | Predominantly entertainment-oriented, including television series, Douyin, and card games; prolonged use during leisure time or sleepless periods; information was largely passively received. | Concerns about operational errors and financial risk, together with low perceived need for advanced functions, restricted further learning. His son provided the device and initial assistance. |
| **P2** | 0.11 | Undergoing rehabilitation following an accident; phone use was affected by dizziness and rehabilitation activities. Actively searched for rehabilitation information and selectively tried advice after personal evaluation. | Able to use WeChat, video, mobile payment, navigation, and basic searching. Learning more complex functions was slower, and assistance was sought from children and friends when necessary. | Mainly used the internet for news, communication with family and friends, and rehabilitation information. Digital services were used selectively, with caution toward online transactions. | Concerns about operational errors and unreliable online information were prominent. Face-to-face services were often preferred. Children and peers provided important learning support. |
| **P3** | 0.42 | Higher frailty level with post-illness impairment of one hand, resulting in reduced phone use and social interaction. Rarely searched actively for disease-related information because of concerns about credibility. | Able to use WeChat, video calls, mobile payment, and simple searches, but physical impairment reduced ease of operation. | Mainly used WeChat, free novels/videos, communication, and basic payment. Posting on social media and overall use declined after illness. | Upper-limb impairment was a major constraint. Children restricted some banking and shopping functions because of fraud concerns, while the participant had limited motivation to learn additional functions. |
| **P4** | 0.07 | Generally favorable health status and active interest in health and rehabilitation information, with attempts to understand its practical application. | Able to use a smartphone and basic computer functions and to actively search for news and health information. Strong willingness to learn was accompanied by difficulties retaining newly learned procedures. | Strong information-seeking orientation, particularly toward technology, politics, military affairs, and health information; relatively prolonged daily use and active searching. | Memory-related difficulty, concerns about fraud, and uncertainty about information quality were the main barriers. Books, written notes, assistance from his son, and senior-learning courses were used as coping resources. |
| **P5** | 0.21 | Reported poorer health and actively searched for rehabilitation and chronic-disease information, selectively trying advice in daily life. | Previous computer experience supported largely self-directed learning. Able to search for information and use navigation, with relatively high confidence in general operations. | Primarily news-, commentary-, and health-information-oriented, with relatively long use duration; also used social communication functions but deliberately avoided financial transactions. | Financial-risk concerns were the major constraint. As children lived far away, most problems were managed independently, with relatively limited external support. |
| **P6** | 0.45 | Higher frailty level. Internet use decreased substantially after illness because of discomfort and intensive rehabilitation. Considerable household and grandchild-care responsibilities also limited health-related digital engagement. | Able to use WeChat, text/voice messaging, mobile payment, and active search and perceived learning new functions as relatively manageable. | Mainly used the internet for news, social communication, and payment. Expressed interest in learning functions such as online appointment booking but had limited time, with further reduction after illness. | Household work, grandchild care, and rehabilitation substantially constrained learning time. Family members frequently completed digital tasks on her behalf, reducing opportunities for independent learning. |
| **P7** | 0.37 | Higher frailty level with concerns about visual decline and memory. Maintained an active offline social life and, after illness, began using an AI application to ask about symptoms and health-related issues. | Able to use calls, WeChat, and small-value mobile payments and to perform simple searches through an AI application installed by her child; several functions remained assistance-dependent. | Low-frequency use centered on communication, small payments, and occasional videos. Digital participation supplemented rather than replaced extensive offline social activity. | Visual discomfort, memory concerns, and beliefs about possible adverse effects of prolonged phone use limited further learning. Family support was strong, although ticketing and appointment-related tasks were often completed by others. |
| **P8** | 0.56 | One of the highest frailty levels. Following illness, mobility, hand dexterity, and cognitive responsiveness declined, accompanied by marked reductions in internet use and social contact. | Before illness, able to use WeChat, payment, online shopping, appointment booking, and searching. Independent operation declined after illness, with occasional caregiver assistance required. | Previously engaged in relatively diverse digital activities; after illness, both frequency and scope narrowed substantially toward essential functions such as calling and limited WeChat use. | Physical, motor, and cognitive limitations were predominant, accompanied by eye strain, distrust of false information, and reluctance to burden others. Family members provided operational assistance. |
| **P9** | 0.06 | Favorable health status and proactive health-management orientation. Actively searched for disease information and used AI to identify hospitals and support healthcare decisions. | Strong digital capability with autonomous use of multiple payment platforms, shopping, food delivery, appointment booking, AI, and multiple devices. | Multifunctional and integrated use across reading, news, entertainment, social communication, and health information, with digital activities closely linked to everyday and health-related needs. | Few major barriers. Most problems were solved independently or through nearby contacts. Prior occupational experience and continued learning provided a strong capability base. |
| **P10** | 0.18 | No prominent personal health-related limitations were described, and little evidence of active digital health management was present. | Very limited literacy and digital capability; able to make conventional phone calls and occasionally watch videos but unable to use WeChat, mobile payment, or other digital services. | Extremely limited use centered on telephone communication, with occasional video viewing; cash and offline routines remained dominant. | Low literacy, memory difficulties, low digital self-efficacy, and limited perceived need jointly constrained learning. Although children had attempted to teach her, she perceived the functions as difficult and unnecessary. |
| **P11** | 0.1 | Little evidence of active digital health management; eye discomfort limited prolonged screen use. | Able to use mobile payment, red packets, and transfers, but unable to actively search, initiate WeChat interactions, or connect independently to Wi-Fi. Capability was highly selective. | Mainly consumed videos and information sent by others while also using calls and mobile payments; broader digital participation remained limited. | Lack of mobile data and dependence on Wi-Fi restricted practical access. Memory difficulties and low learning motivation constrained expansion. Children provided the smartphone, network setup, and initial instruction. |
| **P12** | 0.09 | Generally favorable health status. Actively searched for health information and emphasized the need to judge information before adopting it. | Proficient in basic functions such as WeChat, video, mobile payment, transfers, and searching; some shopping, appointment booking, and more complex tasks were handled by children. | Mainly used the internet for news, social communication, payments, and selective health information. Use was practical, need-oriented, and of relatively limited duration. | Strong concerns about fraud, product mismatch, and suspicious calls; limited interest in unnecessary new functions. Children provided assistance when needed. |
| **P13** | 0.56 | Higher frailty level with pain, visual discomfort, memory decline, and low mood. Interest in internet use decreased markedly after illness. Occasionally encountered health information passively and tried some suggestions, but information-evaluation capability was limited. | Mainly able to make calls and use Douyin; unable to use WeChat messaging, video calls, or mobile payment. Learning and retaining new functions were difficult. | Before illness, spent long periods watching Douyin and military news, largely through algorithmic recommendations; use declined substantially after illness. | Pain, vision, memory, and emotional state were major barriers. She perceived further learning as unnecessary because of age, while family teaching was limited and problems were often handled by others. |
| **P14** | 0.14 | Generally favorable health status and strong health-information-seeking orientation; used AI to obtain information related to cardiac surgery and diabetes. | Strong capability across mobile payment, shopping, appointment booking, banking applications, social media, content creation, and multiple AI tools. | Active exploration involving news, knowledge acquisition, social communication, and multiple everyday services, together with health-information seeking. | Few operational barriers. Main concerns related to online fraud, information overload, and AI accuracy; demonstrated relatively advanced information-evaluation awareness. |
| **P15** | 0.2 | No prominent active health-management behaviors were identified; personal health was not the primary barrier to digital participation. | Mainly able to make calls and use Douyin; limited mastery of WeChat voice, payment, and more complex functions, with recurrent forgetting. | Narrow use centered on short videos and calls; perceived the smartphone as having limited personal value and did not actively extend use to shopping or payment. | Lower educational attainment, memory difficulties, low learning motivation, fraud concerns, and device/battery/data problems jointly constrained participation. A grandchild provided troubleshooting support. |
| **P16** | 0.08 | Self-rated health was good, although eye discomfort limited prolonged viewing. Internet use was minimally connected to health management. | Able only to make calls and watch some Douyin content; did not use WeChat or mobile payment and had limited autonomous digital capability. | Low-frequency use centered on calls and occasional entertainment videos, with continued reliance on cash and offline routines. | Illiteracy, concern about financial errors, low learning motivation, and limited availability of children for teaching were prominent constraints; grandchild care also occupied time. |
| **P17** | 0.12 | No prominent health-related functional limitation was reported. Internet use primarily supported social contact and leisure rather than health management. | Able to use basic WeChat and phone functions and a tablet for chess, but self-described as only “basically able” and unable to perform functions such as online shopping. | Basic-function use centered on communication and chess. Expressed a clear desire to learn additional services such as online shopping. | Lack of sustained teaching and hands-on support was the main barrier. His daughter did not teach shopping because of concerns about impulsive purchases, and he explicitly wanted community-based instruction. |
| **P18** | 0.16 | Generally favorable health status and some engagement with health and wellness information. During hospitalization, deliberately reduced use to protect rest and treatment routines. | Able to use WeChat, payment, transfers, and basic information seeking; still required his daughter’s help for more complex hospital-information functions. New operations were generally learned without major difficulty. | Mainly news, social communication, and health information, with relatively long use duration and some entertainment/emotional-regulation functions; occasional over-engagement occurred. | The main issues were not access but limited capability for some complex services and occasional excessive use. His daughter was an important support source, while substantial offline walking was maintained. |
| **P19** | 0.13 | Macular degeneration and severe visual impairment were the dominant health-related constraints, directly resulting in very low smartphone use. | Continued to use mobile payment but required enlarged text configured by his daughter; most other digital activities were restricted by visual impairment. | Use was largely limited to calling and necessary payment, while leisure remained predominantly offline. | Visual impairment was the primary barrier. Children supported continued basic digital participation through teaching and accessibility adjustments. |
| **P20** | 0.13 | Self-rated health was good, with little evidence of health-related digital management. Resource and capability limitations were more salient than health constraints. | Able only to make WeChat calls and watch videos; unable to use payment, text messaging, or active searching. The smartphone was purchased by his son, but sustained instruction was lacking. | Mainly watched whatever videos appeared and used the phone for calls. Use was low during farm work and increased during hospitalization because of greater free time. | Primary-school education, rural context, low motivation to learn, and limited family instruction constrained further use. Cash remained preferred, and complex digital functions were perceived as cognitively demanding. |
| **P21** | 0.15 | Self-rated health was good, with little active digital health management; prolonged screen use sometimes caused eye discomfort. | Able to use basic WeChat, payment, and video functions but unable to actively search or use more advanced services such as shopping and appointment booking. | High-frequency use, mainly short videos, livestreams, and short-form dramas, showing a clear entertainment orientation and passive content-consumption pattern. | Limited perceived need for additional functions; necessary digital tasks were delegated to children. Learning motivation was low and offline activity was relatively limited. |
| **P22** | 0.08 | Self-rated health was good, with no prominent personal health limitation, but substantial caregiving responsibility for a dependent spouse and paid work constrained available time and energy. | Able only to make calls and watch videos configured by her daughter; did not use WeChat and had very limited autonomous capability. | Low-frequency digital use centered on calls and short videos, with almost no engagement in more complex digital services. | Illiteracy, low digital self-efficacy, fear of fraud, caregiving responsibility, and work demands constituted substantial resource constraints. Problems were mainly resolved through her daughter. |
| **P23** | 0.52 | Higher frailty level following stroke, accompanied by cataracts, memory decline, and a strong focus on rehabilitation. Motivation to learn and broader social contact declined after illness, although some rehabilitation information was still sought. | Able to use WeChat, mobile payment, and read articles; could master basic functions through repeated practice. Shopping, food delivery, and AI remained more dependent on spouse or children. | Mainly used WeChat communication, payment, health articles, and online coordination of offline social activities. After illness, learning of new functions was largely suspended and use became more conservative. | Functional decline and memory problems were important barriers, while a digitally capable spouse provided substantial support. Delegation of complex tasks also reduced motivation for independent learning. |
| **P24** | 0.1 | Proactive health-related digital participation, particularly through the use of AI to obtain rehabilitation guidance for his spouse after stroke; strong ability to integrate health-related resources. | Previous technical occupational background and strong autonomous capability across payment, shopping, food delivery, information searching, and AI. | Digital technologies were broadly integrated into everyday life and family health care, characterized by continued learning, active searching, and multifunctional use. | Few major operational barriers. Occasional assistance was sought from children, while previous technical experience and an enduring learning orientation were the primary enabling conditions. |

**Supplementary material 5**

**Cross-Case Comparison and Persona-Grouping Matrix**

| **Dimension** | **Persona 1 Health-Empowered Integrators** | **Persona 2 Basic-Function Adapters** | **Persona 3 Entertainment-Oriented Users** | **Persona 4 Resource-Limited Users** | **Persona 5 Health-Affected Users** |
| --- | --- | --- | --- | --- | --- |
| **Participants** | P9, P14, P24 | P2, P4, P5, P12, P17, P18 | P1, P21 | P10, P11, P15, P16, P20, P22 | P3, P6, P7, P8, P13, P19, P23 |
| **Health status and self-management** | Proactive health information seeking; active self/family health management | Variable health status; selective, need-based health management | Limited active health management; health status varied | Limited digital health management; health not the primary constraint | Health- and functional limitations; reduced participation after illness |
| **Digital participation foundation and capability** | Stable access; proficient, autonomous, and extensible use | Stable access; basic-to-moderate capability; difficulty with complex functions | Stable access; limited active-search and service-use capability | Basic or assisted access; low autonomy and limited functional skills | Access retained but often reduced or assisted; health-constrained operation |
| **Internet-use orientation and behavioral patterns** | Proactive exploration; multifunctional and health-oriented use | Need-oriented use; selective expansion beyond basic functions | Entertainment-oriented; predominantly content-viewing use | Calls and passive video viewing; minimal digital-service use | Use narrowed after illness; familiar and essential functions prioritized |
| **Constraints and enabling conditions** | Few major barriers; strong self-learning capability with supplementary support | Memory, complexity, and risk concerns; support needed for unfamiliar tasks | Low motivation for functional expansion; family handles other digital tasks | Low education; device/data, learning-opportunity, and support constraints | Health-related functional limitations; reliance on family/caregiver assistance |
| **Dominant cross-case configuration / grouping rationale** | Autonomous, multifunctional digital use combined with proactive health-related use and few major constraints. | Stable access and basic-to-moderate capability, but complex functions, risk concerns, or limited support constrain deeper and more autonomous use. | Basic digital access is retained, but use remains concentrated on entertainment/content consumption with limited expansion to active search or digital services. | Educational, access, learning-opportunity, and support constraints dominate, preventing basic or assisted access from developing into autonomous digital participation. | Physical, sensory, motor, cognitive, or illness-related limitations are the dominant factors narrowing use and increasing reliance on assistance. |
